# Performance and Durability of Porous Asphalt Mixtures Manufactured Exclusively with Electric Steel Slags

**DOI:** 10.3390/ma12203306

**Published:** 2019-10-11

**Authors:** Marta Skaf, Emiliano Pasquini, Víctor Revilla-Cuesta, Vanesa Ortega-López

**Affiliations:** 1 Department of Construction, University of Burgos, 09001 Burgos, Spain; 2 Department of Civil, Environmental and Architectural Engineering (ICEA), University of Padua, 35131 Padua, Italy; emiliano.pasquini@unipd.it; 3 Department of Civil Engineering, University of Burgos, 09001 Burgos, Spain; vrevilla@ubu.es (V.R.-C.); vortega@ubu.es (V.O.-L.)

**Keywords:** steel slag, ladle furnace slag, electric arc furnace slag, porous asphalt, permeable pavement, waste management

## Abstract

Electric arc furnace slag (EAFS) and ladle furnace slag (LFS) are by-products of the electric steelmaking sector with suitable properties for use in bituminous mixtures as both coarse and fine aggregates, respectively. In this research, the production of a porous asphalt mixture with an aggregate skeleton consisting exclusively of electric steelmaking slags (using neither natural aggregates nor fillers) is explored. The test program examines the asphalt mixtures in terms of their mechanical performance (abrasion loss and indirect tensile strength), durability (cold abrasion loss, aging, and long-term behavior), water sensitivity, skid and rutting resistance, and permeability. The results of the slag-mixes are compared with a standard mix, manufactured with siliceous aggregates and cement as filler. The porous mixes manufactured with the slags provided similar results to the conventional standard mixtures. Some issues were noted in relation to compaction difficulties and the higher void contents of the slag mixtures, which reduced their resistance to raveling. Other features linked to permeability and skid resistance were largely improved, suggesting that these mixtures are especially suitable for permeable pavements in rainy regions. In conclusion, a porous asphalt mixture was produced with 100% slag aggregates that met current standards for long-lasting and environmentally friendly mixtures.

## 1. Introduction

In the circular economy, iron and steel stakeholders are trying hard to change the concept of ‘slags as residues’ to ‘slags as co-products’ [1,2]; i.e., valuable by-products from the iron and steel industries that are worth considering in design processes for their reuse and their recycling at levels of 100%, a common practice in other industrial sectors [3,4]. 

The most prevalent process in the production of carbon steel in Spain and Italy is the electric cycle, in which recycled scrap iron is smelted in an electric arc furnace (EAF) and then refined in a ladle furnace (LF). A production method that in Spain alone produces over a million tons of slag every year [5,6]. Although these electric slags present several applications, there is still a significant excess of both materials that continues to be dumped in landfill sites, with the inevitable environmental and landscaping issues. Science, industry, and government alike are, therefore, searching for new alternatives, motivated by environmental concerns, which will decrease the dumping of those by-products [7]. 

EAF slag (EAFS) has a long history as an aggregate in construction materials and other industrial applications after crushing and sieving [8]. Traditionally employed in granular layers, such as subgrades and bases for road and railway embankments [9,10,11], it is a quality gravel for unbound uses, thanks to its high abrasion resistance, roughness, California Bearing Ratio (CBR), and angularity. The features of EAFS are also suitable for use as an aggregate in asphalt mixes for road pavements, [8,12,13]; the high polishing resistance and low abrasion loss of EAFS means that it is in high demand, even for surface layers. The main lines of current research into EAFS cover the manufacture of hydraulic concrete with EAFS as both coarse and fine aggregate [14,15] and, more recently, self-compacting concrete [16,17]. They are yielding remarkable results for mechanical strength, workability, and durability.

The reuse of LF slag (LFS) or secondary slag, a by-product from the basic refining of steel, is less widespread, due to its dusty appearance [18,19]. LFS presents hydraulicity that provides it with slightly cementitious properties [20,21], thus the addition of LF slag is mainly explored when preparing Portland cement mixtures [20,22,23]. The potential of LFS in building and construction research has also been studied, mainly to replace cement and lime in varied applications, such as mortars and concrete [24,25,26], plasterboard [27], and soil stabilization [28], among others. One of the issues related to the reuse of slag is its potential expansion. Electric steelmaking slags usually contain some unstable minerals, predominantly free lime, and magnesia, which undergo transformations in the presence of moisture by hydration and carbonation processes, thus occupying a larger volume than the primary components [29]. These components are usually limited in standards and expansion tests are prescribed for slags when used as a construction material [30]. In addition, European regulations usually specify leaching tests and place restrictions on the eluate content of hazardous substance, to ensure safe reuse of the slags. Still, these requirements refer to a test directly carried out on the loose material, while the application under study in the form of aggregates for bituminous mixtures is far less problematic. It has been proven that “steel aggregates wrapped in a bituminous matrix emit only a small fraction of the leached element” [31]. The use of slags in bituminous mixes has repeatedly been tested and shown to be environmentally safe [32,33,34].

Porous asphalt (PA) is a special type of bituminous mix with a coarse skeleton and few fines, which generates “stone-on-stone contact”, and produces mixtures with high levels of connected air voids [35,36], which explains its good drainage properties due to its porosity. The use of permeable pavements has many environmental and stormwater management benefits [37,38]: it increases safety in wet-weather driving [39], improves water quality after drainage [40], and contributes to comfort in terms of noise reduction [41,42]. 

Almost all conventional bituminous mixes have been manufactured using enormous amounts of natural aggregates. Usually mined from quarries, global consumption of natural aggregates exceeds 30,000 million tons every year [43]. Moreover, their impact is not only a matter of the over-exploitation of limited natural resources, but also concerns the energy consumed and the emissions derived from their mining, crushing, sieving, washing, and transportation. If that trend is to be reversed, the possibility of manufacturing sustainable bituminous mixtures without any natural aggregate or filler, but just with recycled materials, should be explored and validated. The impact reduction in terms of life cycle assessment, studied in the case of mixtures with steel slags, is noticeable in most scenarios [44]. Slags are, however, heavier and will have a negative impact on transport costs and emissions, as well as slightly increasing binder consumption, due to their greater porosity and absorption capacity [45]. However, a 20% reduction in impacts was estimated, when applying similar transport distances to natural aggregates, for asphalt mixtures manufactured with slags [46].

Given this background, the feasibility of using electric steel slags to substitute the ordinary components in porous asphalt will be studied in this research. Its aim is to also take advantage of the granulometric complementarity of these materials, exploring their use without crushing and maximizing their potential combinations in their original state, as some other studies have done [47,48,49]. First, the LFS was used as sand and filler, in substitution of siliceous fine aggregate and cement. Then, the EAFS was also added, as coarse aggregate, replacing the siliceous gravel. 

Tests of all the asphalt mixes in this work were instrumental in determining their mechanical behavior, moisture susceptibility, durability, resistance to permanent deformation, and skid resistance. The ultimate purpose is to prove that it is viable to make porous asphalt mixtures manufactured exclusively with electric steel slags and without any natural aggregates or cement filler, meeting the most relevant requirements and producing a long-lasting and sustainable pavement.

## 2. Materials and Methods 

**Figure 1:**
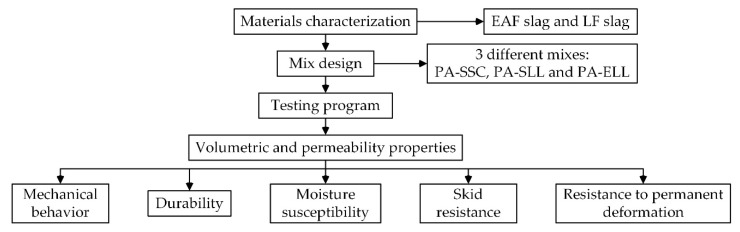
Experimental plan.

This research consists of three phases, which are briefly outlined in Figure 1.

The description of the materials can be divided into the main components of the asphalt mixes: coarse aggregates (2/16 mm), fine aggregates (0.063/2 mm), filler material (under 0.063 mm), and bitumen. The materials employed in this investigation are presented below: siliceous aggregates, EAF and LF slags, cement, and asphalt binder. 

### 2.1. Natural Aggregates, Cement, and Binder

**Figure 2:**
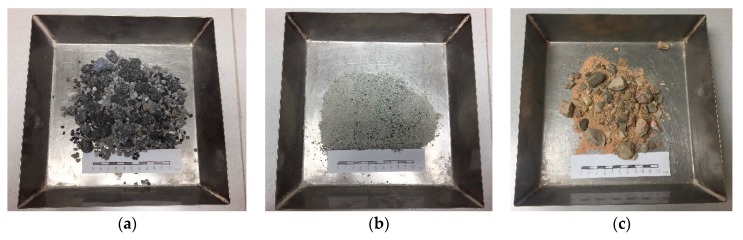
(**a**) EAFS; (**b**) LFS; (**c**) siliceous aggregate. Dimensions in cm.

Natural siliceous aggregates from crushed quartzite from a local quarry were supplied in size fractions of 0/16 mm, as coarse and fine aggregates (Figure 2); their characteristics are summarized in Table 1.

Ordinary Portland cement, type CEM I/42.5 R was employed for comparison in the reference samples, made with standard components. This quality filler is frequently used in combination with siliceous aggregates, which as a mineral filler presents poor adhesion to the binder, due to its acidity. 

The same bitumen, a polymer modified bitumen, PMB 45/80-60, specified in EN 14023 [57], was used for all specimens. This bitumen is produced by a chemical reaction between a hydrocarbon binder and an elastomeric polymer. It is characterized as having a 45/80 dmm penetration and a softening point at 60 °C. The Spanish standard PG-3 [58] requires modified bitumen to manufacture open and porous bituminous mixtures. 

### 2.2. Ladle Furnace (LF) Slag and Electric Arc Furnace (EAF) Slag

The slags used in this investigation are listed in Figure 2. The LF slag is presented as a powdery material with gray and white tones, under 2 mm in size, and the EAF slag as a coarse blackish-gray gravel (2/16 mm in size) aggregate, with some metallic incrustations. Table 1 and Table 2 detail the physical features and the chemical composition of both the LF and the EAF slags, respectively.

**Figure 3:**
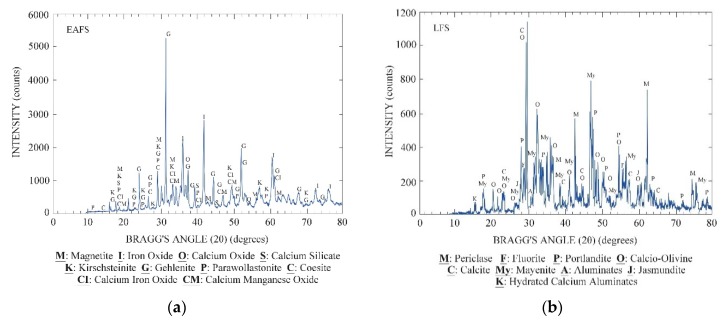
(**a**) EAFS diffractogram; (**b**) LFS diffractogram.

The main mineral fractions of the EAF slag were iron oxides and calcium silicates, accompanied by minor amounts of other oxides and aluminates (Figure 3a). The analysis of the potentially expansive compounds resulted in values under 0.5% for the free lime and under 0.1% in the case of the free magnesia [59]. The LF slag presented periclase, portlandite, and calcium olivine silicates, and minor amounts of lime and reactive aluminates, such as mayenite (Figure 3b). 

Recent investigation on potential expansion that examined the same EAFS in the present research showed negligible expansion (<0.5%), noted after appropriate weathering and curing [30]. However, high delayed swelling was recorded in previous studies on LFS by this research group [28]. As volumetric stability is critical in construction, cautious use of LFS is therefore advised [60]. Some recommendations to promote its use may include: the use of this material in small proportions, wrapped in a bituminous matrix to protect it from moisture, and to use it in flexible and porous matrices, to absorb any eventual expansion [61].

### 2.3. Specimen Preparation

The asphalt mixture was prepared according to the specifications on materials, preparation, and mixing prescribed in EN-12697-35 [62]. According to the manufacturer’s recommendations, polymer-modified bitumen was applied at 160 °C for mixing and at 155 °C for compaction. 

Marshall samples (diameter 101.6 mm, height around 63.5 mm) were manufactured according to EN 12697-30 [63], with the impact compactor, applying 50 blows on each face. Slab samples (410 mm × 260 mm × 40 mm) were manufactured with the roller compactor EN 12697-33 [64], where the loose mixture was compacted to the target air void content, using different levels of compaction energy.

The Marshall specimens were used for the Cantabro, the permeability, and the indirect tensile strength tests. The slab samples were prepared to perform wheel tracking and skid resistance tests. Draindown tests were conducted on uncompacted samples. Every specimen was also subjected to density and void content tests before their final testing. Details on the test methods are provided below.

### 2.4. Mix Design

Three types of mixtures were tested: first, a reference mix, PA-SSC, as a control specimen, manufactured with the conventional materials (siliceous aggregates and cement); second, mix PA-SLL, prepared using siliceous coarse aggregates, with the LFS as fines and filler; and, third, mix PA-ELL that contained the EAFS as coarse aggregate and the LFS as filler and fine aggregate.

In accordance with Spanish Standard PG-3 [58], the particle size distribution of all the mixtures met the PA-11 envelope shown in Table 3. Such a gradation distribution refers to a porous bituminous mixture, with aggregates having 11 mm maximum nominal size and a very thick skeleton, lacking fines and with a large void ratio (>20%). 

In a preliminary mix-design phase, described deeply in Skaf et al. [61], the investigations were focused on selecting the optimum bitumen content (OBC) and the aggregate gradation for SCC and SLL mixtures, both having the same design, mid-band gradation, and filler/binder ratio equal to 1. Some batches of samples were produced with asphalt contents ranging from 4.5% to 6.0% by weight of the total mixture, at 0.5% increments. The final decision was taken based on the performance in the particle loss test (EN 12697-17 [65]), the binder drainage test (EN 12697-18 [66]), and the volumetric properties of the mixtures [61]. 

**Figure 4:**
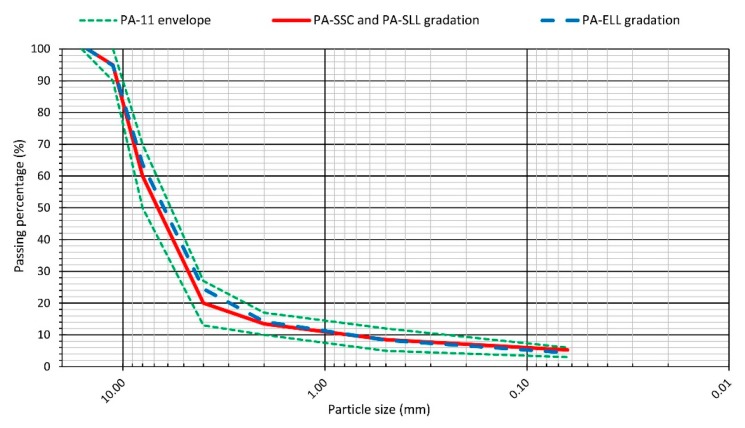
Particle size gradation of the mixtures.

In the next phase, several additional corrections were introduced to this mix design for the ELL mixtures: first, a volumetric correction considering the different densities of the EAF slag coarse aggregates; second, a bitumen correction, due to the higher absorption of the slag; and third, a shift of the gradation towards the finest region of the prescribed band for suitable compaction. The final mix design of all the mixtures can be seen in Table 4, where the dosages of the different components are reported in terms of percentage weight over the total weight of the mixture. The particle size envelope, as well as the mix design of the mixtures, have been also plotted in Figure 4.

### 2.5. Testing Program

All the following tests described below were performed in triplicate on each of the three types of mixes: PA-SSC (conventional reference mix), PA-SLL (siliceous coarse aggregate and LFS fines and filler), and PA-ELL (EAF coarse aggregate and LFS fines and filler).

#### 2.5.1. Volumetric and Permeability Properties 

The volumetric properties were monitored prior to testing each specimen. Thus, based on the mathematical method described in EN 12697-5 and the geometrical method described in EN 12697-6, both the maximum density and the bulk density of the mixtures were assessed. The air void content (AVC) and the voids in the mineral aggregate (VMA) of the specimens were determined according to EN 12697-8.

These results were confirmed by three-dimensional reconstruction from computerized axial tomography (CT). The CT equipment (YXLON, CT Compact, Burgos, Spain) consisted of an X-ray machine, with a 225 Kv/30 mA Yxlon tube. Sections with an interdistance of 0.2 mm were examined to obtain over a thousand images. The pixel size was 0.1244 mm. The data obtained were then processed with the “Mimics” software (10.0, Materialise NV, Leuven Belgium), so that the sequential images captured by the equipment were combined, digitally reconstructing a 3D image that could be studied. Using this technique, pores larger than 170 μm can be quantitatively determined, to obtain information on the structure of the mineral skeleton, as well as the distribution and the number of voids.

The permeability of the mixes was also determined, based on the vertical permeability test defined in EN 12697-19 [67] with a constant head permeameter.

#### 2.5.2. Mechanical Behavior

**Figure 5:**
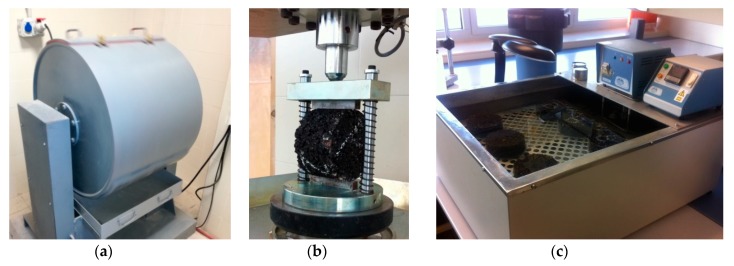
(**a**) Cantabro test; (**b**) ITS test; (**c**) thermostatic bath.

Abrasion Loss (AL), or the wear resistance of the mixtures, is considered the critical feature for asphalt mixes with high void contents [68]. In this case, it was determined by the Cantabro test, which is widely used to assess the abrasion or raveling resistance of these mixtures and has been demonstrated to present the best correlation with the performance and durability of the porous asphalt [69]. In this test, defined in EN 12697-17 [65], each sample is subjected to 300 revolutions in the Los Angeles drum, without steel spheres (Figure 5a). 

Particle loss, PL (%), was then calculated as the weight of the fragmented particles, (*W*_1_ − *W*_2_, g) divided by the original weight of the sample (*W*_1_, g):

(1) $$PL=100\left(W_{1}-W_{2}\right)\;/W_{1}$$

The second parameter to be analyzed was indirect tensile strength (ITS), which was performed as prescribed in EN 12697-23 [70]: the sample was submitted to longitudinal compressive loading, by means of a device that transmits the load onto a vertical plane of the specimen through curved bands placed over the sample (Figure 5b).

The ITS (N/mm^2^) was then calculated from the maximum load at failure, P (N) and the measurements of the sample, h (thickness, mm), and R (base radius, mm):

(2) ITS=P/(π×h×R)

#### 2.5.3. Durability

Durability tests usually consist of comparing some mechanical property (abrasion loss was chosen in this case) of fresh specimens with others after an aging or conditioning process. In this study, three different approaches were used:

- In the aged abrasion loss (AAL) test, the aging process as per ASTM D-7064 [71] consisted in conditioning the samples in an oven for seven days at 60 °C.
- Additionally, in the long-term performance (LTP) test, specimens underwent aging in a controlled atmosphere humidity chamber at 23 °C and 96% humidity for six months, to evaluate the result of bitumen aging on the cohesion of the mixes.
- Cold abrasion loss (CAL) was then evaluated through the procedure proposed by Alvarez et al. [69], by conditioning the samples at a near-freezing temperature of 1 °C over 24 h, to evaluate the stiffness of the binder, the potential brittle fracture, and the susceptibility to cracking of the porous asphalt.

The three durability approaches were evaluated in absolute mean values (*PL_a_*: particle loss- aged, *PL_lt_*: particle loss-long term, *PL_c_*: particle loss-cold) and compared to the results of the fresh tests (*PL*: particle loss-fresh), through “loss increment indices” defined as follows:

(3) $$Aged\;Abrasion\;Loss\;index\;AAL\;index=PL_{a}/PL_{}$$

(4) $$Long‐Term\;Performance\;index\;LTP\;index=PL_{lt}/PL_{}$$

(5) $$Cold\;Abrasion\;Loss\;index\;\;CAL\;index=PL_{c}/PL_{}$$

#### 2.5.4. Moisture Susceptibility 

The water sensitivity of the mixtures was assessed through the procedure described in EN 12697-12 [72], by manufacturing two sets of Marshall samples: the reference group remained at room temperature, and the other group was immersed in 40 °C water for 72 h (Figure 5c). After the above-mentioned curing process, the specimens were subjected to the well-known ITS test (Figure 5b), according to EN 12697-23 [70]. 

Specimen resistance to moisture damage was evaluated through the indirect tensile strength ratio (ITSR), which compares the results of both the wet group (*ITS_w_*) and the standard dry group (*ITS_d_*):

(6) $$ITSR\;\left(\%\right)=100\;\times \;ITS_{w}/ITS_{d}$$

#### 2.5.5. Skid Resistance

The skid resistance of the surface courses depends on the adhesion tire-pavement and is directly linked to accident rates [73]. The slipping resistance of a pavement surface depends both on its macro and on its microtexture.

**Figure 6:**
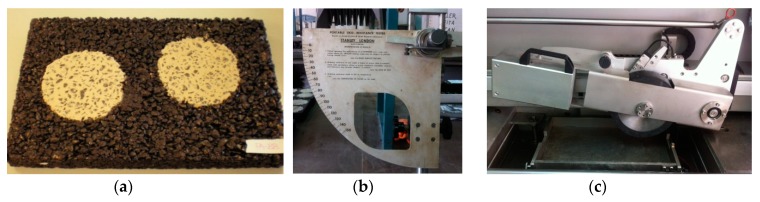
(**a**) Macrotexture; (**b**) British pendulum; (**c**) wheel tracking device.

Here, the macrotexture was measured by the volumetric patch technique, as described in EN 13036-1 [74], where a certain volume of very fine sand is spread out in a circle, so that it fills the hollows and then the average diameter of the circle that is formed is determined (Figure 6a). The mean texture depth (MTD, mm) is related to the volume of sand introduced (V, mm^3^) and the mean diameter of the circle (D, mm):

(7) $$MTD=\;4V/\pi \;D^{2}$$

Subsequently, the microtexture was assessed with the portable British pendulum test (Figure 6b), as per EN 13036-4 [75], in which a rubber-coated pad slides along the surface of the sample and its friction is reflected in a graduated reduction in the length of the oscillation: the higher the friction value, the higher the British pendulum number (BPN).

These BPN tests were performed on two specimens of each kind: firstly, fresh specimens and then others, which had been slightly polished, mainly to remove the remaining mastic on the surface of the recently laid asphalt, in simulation of the effects of initial traffic loads, which can produce inconsistent results. Three separate locations were tested on each slab, then the average of all three BPN values was obtained and, finally, the results were all adjusted to a BPN equivalent to 20 °C. 

#### 2.5.6. Resistance to Permanent Deformation

Resistance to rutting was evaluated through a computerized wheel tracking machine (Figure 6c), which met the requirements of EN 12697-22 [76]. The experimental conditions were as follows: temperature of 60 °C, a rubber tire (200 mm diameter and 50 mm width) with a pressure of 70 N, and at a speed of 53 passes/min. 

The dynamic stability (*DS*, wheel passes/mm) was calculated by the following equation:

(8) $$DS=15N/(d_{60}-d_{45})$$

where N is the wheel speed, 53 passes/min; and, d_45_, d_60_ are vertical deformations at test times of 45 and 60 min.

## 3. Results and Discussion

### 3.1. Volumetric Properties

The volumetric properties results of every mix tested are shown below, in Table 5. 

Both the maximum density and the bulk density obtained for the PA-SSC and the PA-SLL mixes were similar, which is consistent with the fact that the LFS and the siliceous aggregate have similar densities and the same mix design was used in both cases. Due to the high density of the EAFS, mix PA-ELL, manufactured with this aggregate, showed both a higher maximum density and a higher bulk density than the previous mixes. 

**Figure 7:**
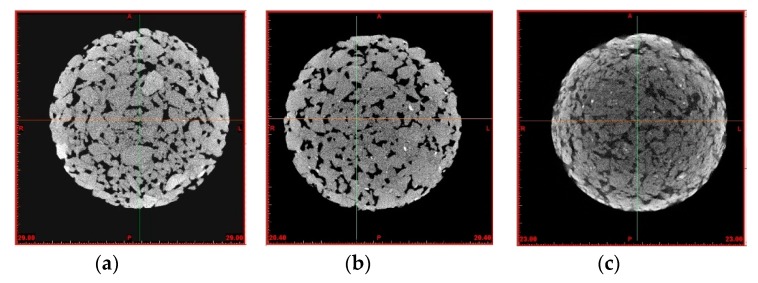
Computed tomography (**a**) PA-SCC; (**b**) PA-SLL; (**c**) PA-ELL.

Regarding the AVC, all the mixtures presented values over 20%, which are required for the Porous Mixtures [58]. However, despite the efforts in the mix design to enhance compaction of the 100% slag mixtures, an increase of about 3% in the void content was observed in mix PA-ELL in the laboratory, which was later corroborated by the computed tomography images (Figure 7). These voids were attributed to the greater angularity and sphericity of the EAFS aggregates when compared with the siliceous aggregates [77]. This circumstance agreed with other references to difficulties with the workability and the compactibility of the asphalt mixes with high contents of slags, which resulted in higher voids [13,78,79,80]. Any increase in the air void content is usually a critical aspect when incorporating a residual product in construction materials [81,82,83]. 

Permeability, as has been shown, is closely related to AVC and VMA values [42,84,85]. As might be expected, the mean results of the tests were very similar in the first two mixtures, with similar void contents, and the introduction of the EAF slag increased their permeability [86], reaching optimum draining performance, higher than 10^−1^ cm/s [87]. 

### 3.2. Mechanical Behavior

In terms of particle loss, as shown in Table 6, every mixture largely met the standard requirements, which have to be lower than 20% in the Spanish standards for the heaviest traffic. Standards in other countries require maximum particle loss values varying from 15% to 30% [88].

Abrasion loss values slightly increased in mix PA-SLL when using the LFS as sand and filler. These losses, explained by the higher binder absorption of the LFS, was noticeable in the mix design phase after the binder drainage test [61]. The higher absorption values mean that the bitumen film covering the coarse particles was thinner, which reduced the wear resistance of mix PA-SLL with respect to mix PA-SCC. 

Moreover, the worse response of mix PA-ELL with 100% slag can be explained by the higher void content that makes them more prone to raveling. A direct correlation between voids and the corresponding abrasion resistance is evident in the literature, in particular for porous asphalt [83,89,90].

Good resistance to shear stress was obtained from the indirect tensile strength tests and close results were observed among the different mixtures, which inferred a good cohesion of the mixes, suitable tensile performance, and good cracking resistance of the slag mixtures, notwithstanding the higher AVC.

### 3.3. Durability

**Figure 8:**
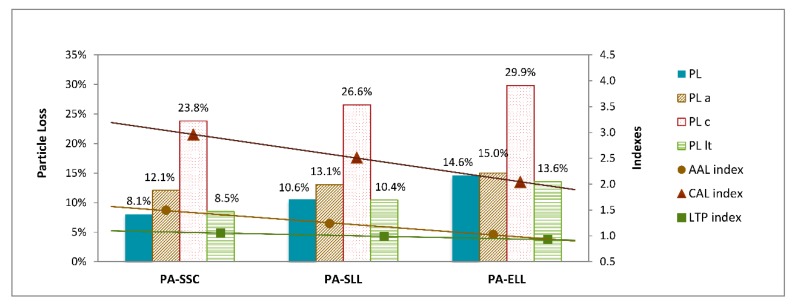
Mixture durability.

**Figure 9:**
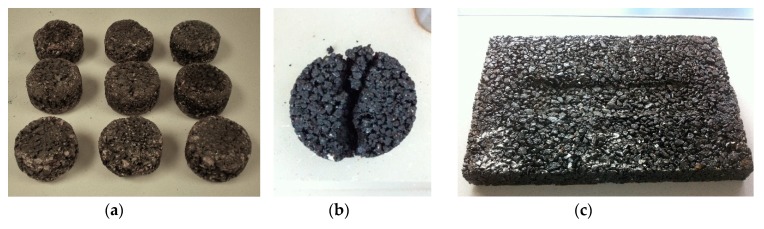
(**a**) Samples after the durability tests; (**b**) specimen after the moisture susceptibility test; (**c**) slab sample after the wheel tracking test.

Figure 8 summarizes the average results of particle loss and the comparisons between the indexes of the assorted durability tests on the bituminous mixtures that were designed. As can be observed in Figure 9a, some samples were subjected to different durability tests. 

In a preliminary analysis, a clear increase in particle loss can be detected in all the samples subjected to both the AAL and the CAL procedures, while the LTP curing hardly affected the material behavior. Regarding the absolute terms, the influence of incorporating the slags in the porous asphalt mixes, as can be observed, followed the same trend, regardless of the curing process. With respect to the reference mix PA-SSC, particle loss increased with increasing amounts of LF slag (PA-SLL) and further decomposition was observed in the case of mix PA-ELL containing only slags. 

However, the aged abrasion loss index showed a decrease in the rate of slag incorporation, from 1.50–1.24–1.03, which indicated that the effect of aging was lower when LFS was used and even lower in mixtures made with LF and EAF slags. The 100% slag mixture was the least susceptible to the aging of the binder, as other researchers also found [13], which ensures a more cohesive mixture and better binder-particle adhesion.

Similar observations can be repeated when the CAL index is considered, revealing that the incorporation of both LFS and EAFS improved the thermal susceptibility of the mixes. The CAL index was reduced from 2.94 in mix PA-SSC to 2.51 in mix PA-SLL and to 2.05 in mix PA-ELL, showing a clear descent in the comparative evolution of their resistance to cold temperatures. Moreover, ASTM D-7064 [71] limits the individual specimen loss to 50% for the abrasion values of single specimens and to 30% for the average mixture results. In this case, every sample easily fulfilled those requirements when tested, presenting an average particle loss after aging lower than 15% in the worst case and individual values of below 20% in all cases; results which, therefore, validated the good performance of all the mixtures. 

### 3.4. Moisture Susceptibility 

**Figure 10:**
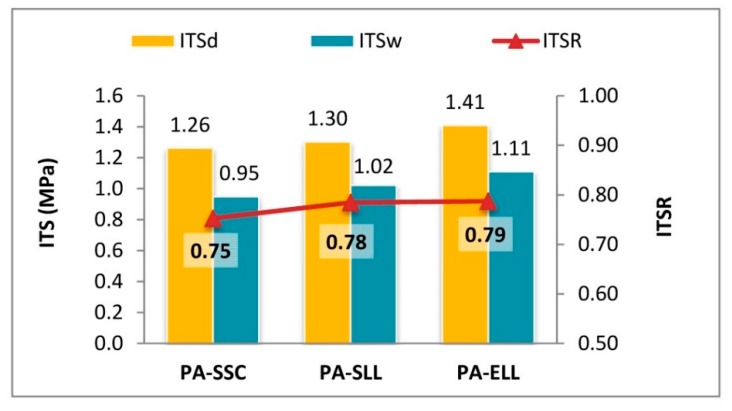
Moisture susceptibility.

Regarding the results of the ITSR, the performance in the mixtures is shown in Figure 10. The reference mix had the worst ITSR coefficient value, while the incorporation of the slag in the mixes led to a slight improvement in the results. Regulations usually require minimum ITSR values among 70% and 80% [91]. Even so, some experts think that this methodology may not be applicable for high void content mixtures [35,91] and that these limits are too strict for this type of materials. Beyond these preliminary considerations, it may be said that the PA-SLL mixes obtained similar results to the PA-SCC mix. These results were similar in both the ITS after wet conditioning (*ITS_w_*) and in the ITSR. It may, therefore, be affirmed that the addition of LF slag formed a quality mastic for manufacturing comparable to other conventional materials. 

When incorporating EAF slag as a coarse aggregate (Figure 9b), the results in *ITS_w_* and ITSR were improved, which is consistent with many studies containing references to moisture sensitivity in asphalt mixes with steel slags [92,93]. The good affinity with the bitumen and the rougher texture of the EAFS is likely to have enhanced adherence and cohesion, counterbalancing the effects of higher AVC.

### 3.5. Skid Resistance

Regarding the microtexture, British pendulum numbers above 50–55 are considered appropriate for safe driving on high speed roads [94]. Therefore, as can be observed in Table 7, the resulting values of the BPN can be considered very good in the fresh state and good for the aged state, both for the standard mixtures and for those incorporating LFS. 

Moreover, when introducing the EAFS as coarse aggregate, the pendulum test resulted in very high values of the BPN, indicating excellent friction of the slag pavements. In addition, the superior microtexture compared to pavements made with coarse siliceous aggregate was maintained over time, yielding results that remained five points higher than the scores of the other mixtures. In the macrotexture analysis, the PG-3 [58] recommends, in the case of porous pavements, that the average depth of the footprint defined by the volumetric test must be higher than 1.5 mm, for a rough texture that favors the lateral expulsion of water from beneath the tire. That minimum value was exceeded in all cases, although in the standard mixes the results were tight, because the finest porous asphalt was chosen for the mix design, with a very small maximum aggregate size that is not conducive to water expulsion. The incorporation of the slag increased the void content and improved the macrotexture in both the LFS and the EAFS specimens, as shown in Table 7.

### 3.6. Resistance to Permanent Deformations

As a preliminary comment in the discussion of the results, it may be noted that the wheel-tracking test was designed to determine rutting resistance in dense mixtures. The results obtained through laboratory tests correlated very well with the behavior of these mixtures, but the validity of that test to characterize open and porous mixtures is still under discussion [69]. In fact, that test is not usually prescribed for porous asphalt, so there are very few available reference values. However, the results (Table 8) can be useful for comparative purposes, with the object of evaluating the effect of slag aggregates on the permanent deformation resistance.

Every parameter of the rutting resistance was improved in the 100% slag mixes compared to the reference mixtures under analysis. Moreover, the Japanese Road Association [95] prescription for porous asphalt is a DS that exceeds 3000 passes/mm. In Spain, standard NLT-173 [96] requires a deformation rate (v) of under 15 µm/min for dense graded asphalt mixtures. Both prescriptions are only fulfilled in the 100% slag porous mixtures. 

A significant improvement in rutting resistance could be detected when testing the sustainable slag mixtures (Figure 9c). The mixes prepared with EAF slag were stiffer and resisted permanent deformation better than the siliceous mixtures, which was consistent with the previous test results and the high angularity of the EAFS as a coarse aggregate with better aggregate interlocking action, as others have demonstrated [97]. This behavior was also consistent with other investigations on the effects of slag incorporation in porous asphalt mixes [91,98]. 

## 4. Conclusions 

Our research has been focused on a study of the feasibility of producing porous asphalt mixtures exclusively containing steel slag aggregates (using neither natural aggregates nor fillers). Electric arc furnace (EAF) and ladle furnace (LF) steel slags were used to replace coarse (>2 mm) and fine (<2 mm) aggregate fractions, respectively. Traditional porous asphalt was selected as the reference mixture for comparative purposes. Based on the main findings arising from the comprehensive experimental program, the following basic conclusions can be summarized:

- Steelmaking slag mixtures were more porous and permeable than the standard mixtures. The high roughness and sharpness of the EAF slag complicated compaction and created mixtures with higher air void contents, even if still meeting the technical specifications.
- In general, abrasion loss results fulfilled the standards for the heaviest loads, but introducing the slags yielded a slightly worse performance than the conventional mixes, which could be due to the increment in porosity of the slag mixes.
- The selected durability indexes were enhanced with the incorporation of slags, making these pavements less susceptible to aging and to thermal cracking.
- The presence of steel slag aggregates, rather than leading to a reduction of the water sensitivity of the mixtures, even improved it. In particular, the mixture prepared with both EAF and LFS showed similar or even improved performance in comparison with the reference mix, in all likelihood due to the rougher texture of the EAFS, which improved adhesion and the affinity with the bitumen, counterbalancing the effects of a higher void content.
- The slag pavements showed excellent skid resistance. Their higher permeability and rougher texture meant that they were very appropriate for rainy regions.
- The specific features of the EAFS as a coarse aggregate enhanced the pavement resistance to permanent deformation.

Although there are still some issues to overcome, such as compactability refining, and specific aspects of economic sustainability, we hope that the results reported here will encourage further research on this topic, to promote the viability of manufacturing 100% sustainable, high-quality porous bituminous mixtures.

## Figures and Tables

**Table 1:** Physical properties of the siliceous aggregate, the EAFS, and the LFS.

Characteristic	Test Method	Natural Agg. (0–16 mm)	EAFS (2–16 mm)	LFS (0–2 mm)	Technical Requirements
Bulk Density	EN 1097-6 [50]	2.74 g/cm^3^	3.60 g/cm^3^	2.83 g/cm^3^	-
Water Absorption	EN 1097-6 [50]	1.5 %	2.1%	0.4%	-
Fineness modulus	EN 933-1 [51]	4.2	-	2.9	-
Sand Equivalent (SE)	EN 933-8 [52]	78%	98%	50%	>50%*
Los Angeles (LA)	EN 1097-2 [53]	20%	23%	-	<25–20–15%**
Polished Stone Value (PSV)	EN 1097-8 [54]	52%	56%	-	>56–50–44%**
Flakiness index	EN 933-3 [55]	18%	3%	-	<20%
Crushability index	EN 933-5 [56]	100%	100%	-	100–90%**

* for the combined fraction ** depending on the road category (i.e., highways, major roads, minor roads).

**Table 2:** Chemical composition of the slags under study.

Component	CaO	SiO_2_	MgO	Al_2_O_3_	Fe_2_O_3_	MnO	CO_2_	Others
EAFS wt%	27.7	19.1	2.5	13.7	26.8	5.4	-	4.8
LFS wt%	56.7	17.7	9.6	6.6	2.2	-	1.3	5.9

**Table 3:** Particle size envelope of the PA-11 porous asphalt mixture.

**Size (mm.)**	16	11.2	8	4	2	0.5	0.063
**% passing**	100	90–100	50–70	13–27	10–17	5–12	3–6

**Table 4:** Final mix design.

Element	Size (mm)	PA-SSC		PA-SLL		PA-ELL	
		Material	Wt.%	Material	Wt.%	Material	Wt.%
Coarse aggregate	16–11.2	Siliceous	4.8%	Siliceous	4.8%	EAFS	5.0%
	11.2–8	Siliceous	33.2%	Siliceous	33.2%	EAFS	29.7%
	8–4	Siliceous	38.0%	Siliceous	38.0%	EAFS	37.6%
	4–2	Siliceous	6.2%	Siliceous	6.2%	EAFS	9.9%
Fine aggregate	2–0.5	Siliceous	4.7%	LFS	4.7%	LFS	5.6%
	0.5–0.063	Siliceous	3.1%	LFS	3.1%	LFS	3.8%
Filler	<0.063	Cement	5.0%	LFS	5.0%	LFS	4.2%
Binder	-	PMB 45/80-60	5.0%	PMB 45/80-60	5.0%	PMB 45/80-60	4.2%

**Table 5:** Volumetric properties: Average and standard deviation values.

Feature	Test	PA-SSC	PA-SLL	PA-ELL
Bulk density (g/cm^3^)	EN 12697-6	2.00 (0.04)	1.99 (0.03)	2.34 (0.04)
Maximum density (g/cm^3^)	EN 12697-5	2.54	2.57	3.09
Air voids (%)	EN 12697-8	21.1 (1.3)	21.7 (1.3)	24.3 (0.7)
	CT	20.5	21.1	25.2
Voids in the Mineral Aggregate (%)	EN 12697-8	30.9	31.4	33.9
	CT	30.2	31.1	34.9
Permeability (cm/s)	EN 12697-19	9.01 × 10^−2^	9.04 × 10^−2^	1.51 × 10^−1^

**Table 6:** Mechanical behavior.

Feature	Test	PA-SSC	PA-SLL	PA-ELL
Abrasion loss (AL)	Void content (%)	19.8	20.0	24.1
	Particle loss, PL (%)	8.06 (1.44)	10.57 (2.02)	14.62 (2.38)
Indirect tensile strength (ITS)	Void content (%)	20.9	20.7	23.3
	Maximum load (N)	12.96	13.53	14.66
	ITS (N/mm^2^)	1.26 (0.03)	1.31 (0.09)	1.41 (0.11)

**Table 7:** Skid resistance.

Feature		PA-SSC	PA-SLL	PA-ELL
Microtexture	BPN fresh	61	61	77
	BPN polished	54	54	59
Macrotexture	Void content (%)	18.5	21.5	25.4
	MDT (mm)	1.53	1.76	1.89

**Table 8:** Wheel-tracking test results.

Feature	PA-SSC	PA-SLL	PA-ELL
Mean void content of the samples	19.53%	20.90%	22.91%
Linear slope (mm/10^3^ cycles)	0.58	0.98	0.24
Dynamic stability, DS (passes/mm)	2500	2000	3500
Deformation rate, v (µm/min)	16	20	12
Rut depth at 4000 cycles, d4000 (mm)	2.6	2.8	2.4
